# Predictors of pacing-induced cardiomyopathy in patients undergoing AV nodal ablation: insights from a Delphi process and retrospective cohort study

**DOI:** 10.1093/ehjopen/oeag102

**Published:** 2026-06-29

**Authors:** Nikola Kozhuharov, Anna Maria Gstoehl, Peter Calvert, Ioanna Koniari, Ali Najm, Mark T Mills, Kaung Thu, Panagiotis Xydis, Raphael Usteri, Agnieszka Babinska, Andreas Haeberlin, Tobias Reichlin, Christian Sticherling, Haran Burri, David Justin Wright, Archana Rao, Dhiraj Gupta

**Affiliations:** Department of Cardiology, Inselspital, Bern University Hospital, University of Bern, Freiburg Strasse 18, Bern 3010, Switzerland; Department of Cardiology, Inselspital, Bern University Hospital, University of Bern, Freiburg Strasse 18, Bern 3010, Switzerland; Department of Cardiology, Liverpool Heart & Chest Hospital, Thomas Drive, Liverpool L14 3PE, UK; Liverpool Centre for Cardiovascular Science, University of Liverpool, 6 West Derby Street, Liverpool L7 8TX, UK; Department of Cardiology, Liverpool Heart & Chest Hospital, Thomas Drive, Liverpool L14 3PE, UK; Liverpool Centre for Cardiovascular Science, University of Liverpool, 6 West Derby Street, Liverpool L7 8TX, UK; Department of Cardiology, Liverpool Heart & Chest Hospital, Thomas Drive, Liverpool L14 3PE, UK; Liverpool Centre for Cardiovascular Science, University of Liverpool, 6 West Derby Street, Liverpool L7 8TX, UK; Department of Cardiology, Liverpool Heart & Chest Hospital, Thomas Drive, Liverpool L14 3PE, UK; Liverpool Centre for Cardiovascular Science, University of Liverpool, 6 West Derby Street, Liverpool L7 8TX, UK; Department of Cardiology, Liverpool Heart & Chest Hospital, Thomas Drive, Liverpool L14 3PE, UK; Liverpool Centre for Cardiovascular Science, University of Liverpool, 6 West Derby Street, Liverpool L7 8TX, UK; Department of Cardiology, Liverpool Heart & Chest Hospital, Thomas Drive, Liverpool L14 3PE, UK; Liverpool Centre for Cardiovascular Science, University of Liverpool, 6 West Derby Street, Liverpool L7 8TX, UK; University Heart Center, University Hospital Basel, Petersgraben 4, Basel 4031, Switzerland; 1st Department of Cardiology and Angiology, Silesian Center for Heart Diseases, Marii Skłodowskiej-Curie 9, Zabrze 41-800, Poland; Department of Cardiology and Electrotherapy, Faculty of Medical Sciences in Zabrze, Medical University of Silesia, 15 Poniatowskiego Street, Katowice 40-055, Poland; Department of Cardiology, Inselspital, Bern University Hospital, University of Bern, Freiburg Strasse 18, Bern 3010, Switzerland; Department of Cardiology, Inselspital, Bern University Hospital, University of Bern, Freiburg Strasse 18, Bern 3010, Switzerland; University Heart Center, University Hospital Basel, Petersgraben 4, Basel 4031, Switzerland; Cardiac Pacing Unit, Cardiology Department, University Hospital Geneva, Rue Gabrielle-Perret-Gentil 4, 1211 Geneva, Switzerland; Department of Cardiology, Liverpool Heart & Chest Hospital, Thomas Drive, Liverpool L14 3PE, UK; Department of Cardiology, Liverpool Heart & Chest Hospital, Thomas Drive, Liverpool L14 3PE, UK; Department of Cardiology, Liverpool Heart & Chest Hospital, Thomas Drive, Liverpool L14 3PE, UK; Liverpool Centre for Cardiovascular Science, University of Liverpool, 6 West Derby Street, Liverpool L7 8TX, UK

**Keywords:** Pacing-induced cardiomyopathy, Pace-and-ablate, Delphi process, Risk predictors

## Abstract

**Aims:**

Pacing-induced cardiomyopathy (PICM) is a recognized complication of pace-and-ablate therapy. Early identification of patients at risk may allow tailored strategies, including upfront biventricular or conduction system pacing.

**Purpose:**

This study aimed to identify predictors of PICM in patients undergoing pace-and-ablate therapy, combining expert consensus with clinical validation.

**Methods and results:**

A three-round Delphi process involving 12 electrophysiologists prioritized candidate predictors of PICM. Consecutive patients undergoing atrioventricular node ablation with non-cardiac resynchronization therapy (CRT) devices at Liverpool Heart and Chest Hospital (2015–2019) were retrospectively analysed. The primary endpoint was PICM, defined as ≥10% reduction in left ventricular ejection fraction (LVEF) to <50%, adjudicated by two independent cardiologists. Cox regression was used to identify independent predictors. The Delphi process prioritized baseline LVEF, intrinsic and paced QRS duration, RV lead position, and key comorbidities. Among 658 ablation patients, 323 (median age 76 years, 67% female) had non-CRT devices. Over a median follow-up of 192 days (IQR, 63–1003 days), 33 patients (9.8%) developed PICM. In multivariable analysis, RV free wall vs. septal lead position [hazard ratio (HR) 4.896, 95% confidence interval (CI) 1.263–18.973, *P* = 0.022], intrinsic QRS duration (HR 1.247 per 10 ms, 95% CI 1.064–1.462, *P* = 0.006), independently predicted PICM. Exploratory analyses suggested that higher intrinsic QRS duration was associated with increased PICM risk; however, these findings were based on a small number of events and should be interpreted with caution.

**Conclusion:**

Intrinsic QRS duration and free wall RV lead position independently predict PICM in pace-and-ablate patients. These findings support early risk stratification and may guide personalized pacing strategies.

## Graphical Abstract

**Graphical Abstract:**
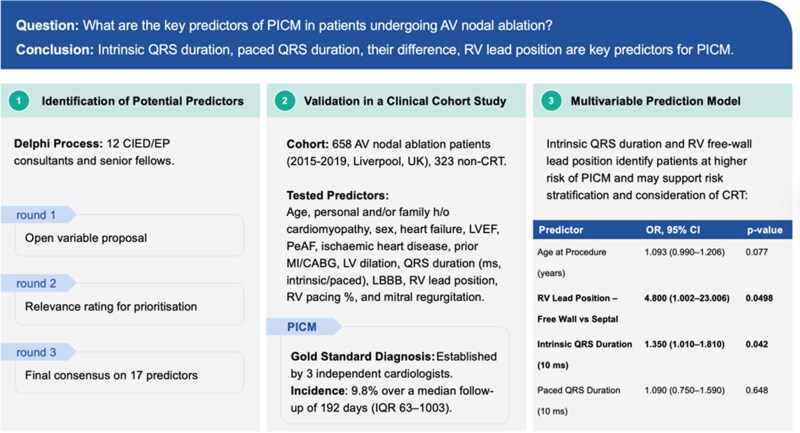

What is new?

- This is the first study to combine expert consensus and clinical validation to identify predictors of PICM specifically in pace-and-ablate patients.
- In a large non-CRT cohort, intrinsic QRS duration and right ventricular free wall lead position emerged as independent predictors of PICM.
- Exploratory analyses suggested that longer intrinsic QRS duration may identify higher-risk subgroups, although these findings are based on a small number of events and require prospective validation.
- Free wall RV lead position carried a nearly five-fold higher risk than septal pacing, underscoring the importance of implantation strategy.
- These results provide a practical basis for early risk stratification and personalized pacing in AF patients treated with AV node ablation.

## Introduction

The pace-and-ablate strategy is a well-established treatment of atrial fibrillation (AF) in patients who are either unsuitable for or have not responded to rhythm control therapies.^1–3^ In certain populations, such as elderly patients with heart failure and cardiac amyloidosis, where pulmonary vein isolation tends to have particularly high recurrence rates, the pace-and-ablate approach may be even preferred as a first-line interventional therapy.^4–6^ As a definite treatment, it ensures reliable ventricular rate control and regularization,^7–10^ together with bradycardia management, thereby contributing to improved prognosis. In heart failure patients with persistent atrial fibrillation, this approach improves survival, reduces heart failure and all-cause rehospitalizations, and enhances quality of life.^3,11–16^

Pacing-induced cardiomyopathy (PICM) remains a major limitation of pace-and-ablate therapy. PICM is characterized by a decline in left ventricular systolic function, which can lead to symptomatic heart failure and reduced survival.^3,16–19^ Often, PICM diagnosis is preceded by acute heart failure, which is associated with high mortality, morbidity and cost.^5,20^ Upfront cardiac resynchronization therapy (CRT) by biventricular pacing (BiVP) or conduction system pacing may mitigate this risk,^3,21,22^ but routine implantation in all patients remains a challenge due to longer procedures, increased complexity, higher costs, and resource constraints. CRT upgrades after the onset of PICM may be technically challenging and are associated with inferior outcomes.^23–25^

While several studies have evaluated predictors of PICM in patients receiving right ventricular pacing, findings are inconsistent, and no widely accepted risk model exists.^17–19^ Importantly, no data specifically address predictors of PICM in pace-and-ablate patients, despite their distinct clinical profile and the potential benefit of early risk stratification.

We therefore conducted a structured Delphi process among electrophysiology specialists to identify candidate predictors of PICM in pace-and-ablate patients, followed by validation in a retrospective cohort. This consensus-driven approach aimed to provide the basis for future risk stratification models and inform individualized pacing strategies in this population.

## Methods

### Study design and population

Predictor selection was performed using a modified Delphi process involving electrophysiology experts from tertiary hospitals across Switzerland (Bern, Geneva, and Basel), Poland (Zabrze), and the UK (Liverpool). The Delphi process results were then validated using retrospectively collected data on consecutive patients undergoing AV node ablation at the Liverpool Heart and Chest Hospital between January 2015 and December 2019. Baseline LVEF was defined as the most recent echocardiographic assessment prior to AV node ablation. The median time between baseline echocardiography and ablation was 323 days (IQR 127–589 days). Patients with CRT (exclusively BiVP in this dataset) or conduction system pacing (including His bundle pacing) at baseline were excluded. Follow-up extended until the development of PICM, device upgrade, death, or last routine clinical contact. Exposure variables were assessed retrospectively from clinical records, including ECG parameters from standard 12-lead ECGs and device- and procedure-related variables from procedural reports and imaging. All data were fully anonymized before analysis. The study followed the principles of the Declaration of Helsinki and received ethics approval (IRAS ID 314643).

### Delphi process for predictor selection and predictor definition

A three-round modified Delphi process was conducted to identify and prioritize candidate predictors of PICM, following established methodologies.^26^ The Delphi process was designed to complement existing literature by incorporating expert clinical judgement in a field with limited and heterogeneous data, particularly in pace-and-ablate populations. In the first round, participants suggested potential clinical and procedural variables. In the second round, all proposed variables were rated for inclusion in an online survey. For pragmatic categorization, priority levels were assigned based on the proportion of experts who voted for inclusion. Variables with ≥75% agreement were assigned priority 1, 50–74% as priority 2, and <50% as priority 3. In the final round, variables with priorities 2 and 3 were reconsidered for exclusion. Between rounds, results were shared with the panel, and a structured discussion via email was encouraged to refine variables selection. Variables supported by the majority were retained, forming the final consensus set for analysis. One of the variables retained was RV lead position—classified as apical, septal, or free wall based on procedural fluoroscopy and post-procedural chest X-ray, as documented in clinical reports. Septal positioning was defined by a leftward orientation in the left anterior oblique view and a posterior location in the right anterior oblique view, consistent with interventricular septal placement. Apical leads were identified by an inferior and anterior position, while free wall leads were defined as non-septal, non-apical positions along the lateral RV wall. Free wall leads were defined as leads located along the lateral or anterolateral right ventricular wall, characterized by a rightward orientation in the left anterior oblique view, inconsistent with septal or apical placement. Where additional imaging (e.g. cardiac CT) was available and deemed clinically relevant, it could be considered. However, this was not protocolized. Septal position was used as the reference category in binary RV lead position Cox regression analyses, as it represents the intended target for standard RV lead placement and provides a consistent comparator for other lead locations.

### Outcome definition and adjudication

The primary outcome was the development of PICM, defined as an absolute reduction in LVEF of at least 10% to a value below 50% on follow-up echocardiography.^17^ Echocardiographic data were obtained from studies performed as part of routine clinical care. Echocardiographic follow-up was not performed at predefined intervals but based on clinical judgement, typically in patients with new or worsening symptoms suggestive of heart failure. Echocardiography dates were systematically captured in the dataset only when PICM was suspected. Patients with short follow-up at the implanting centre were often discharged to local care. However, clinically relevant cases with suspected PICM would generally have been referred back to Liverpool Heart and Chest Hospital, as it is the regional centre performing CRT upgrades. The diagnosis of PICM as the primary endpoint was adjudicated by two independent cardiologists based on all available clinical, device, and imaging data (also including response to CRT upgrade). In the event of disagreement, a third cardiologist participated in the review, and a consensus was reached through discussion. Outcome adjudication was not performed blinded to exposure variables. Cases lacking a complete diagnostic workup or sufficient follow-up were classified as ‘likely PICM’ if no other plausible cause for significant LVEF decline was identified.[16] The primary analysis was based on the composite endpoint of definite and likely PICM. Sensitivity analyses restricted to definite PICM cases were performed to assess the robustness of the findings.

### Statistical analysis

Continuous variables were presented as medians with interquartile ranges (IQR) and compared using the Mann–Whitney *U* test. Categorical variables were expressed as counts and percentages and compared using the chi-squared or Fisher’s exact test, as appropriate. Survival free of PICM during follow-up was plotted in Kaplan–Meier curves, and the log-rank test was used to assess differences between groups. Variables with a *P*-value <0.10 in univariable Cox regression were entered into multivariable models to identify independent predictors of PICM. Hazard ratios (HR) with 95% confidence intervals (CI) were reported. This approach was applied to a predefined set of clinically relevant variables identified through the Delphi process. To address collinearity, highly correlated variables (e.g. difference between paced and intrinsic QRS) were not included in the same model. Proportional hazards were evaluated using Schoenfeld residuals (global and covariate-specific). To further reduce overfitting, model size was limited relative to the number of events and parsimonious sensitivity models were performed. Internal validation of the multivariable Cox regression model was performed using bootstrap resampling (300 resamples), with assessment of optimism-corrected discrimination and calibration.

Furthermore, we performed a fragility sensitivity analysis for RV lead position by repeating the multivariable Cox model after reclassifying 1, 2, or 3 PICM cases from the RV free wall group to the septal group.^27^ For continuous independent predictors in multivariable models, additional analyses were performed to explore cut-offs with potential prognostic relevance, defined by higher HRs and statistical significance in univariable Cox regression. For each cut-off, sensitivity, specificity, PPV, and NPV, and the number of patients ruled in, were calculated, and 95% CIs for these proportions were estimated using Wilson score intervals with continuity correction. A two-sided *P*-value <0.05 was considered statistically significant. Analyses were performed using SPSS version 28.0 (IBM Corp., Armonk, NY, USA) and R version 4.5.2 (R Foundation for Statistical Computing, Vienna, Austria; survival and ggplot packages).

## Results

### Delphi process

In the first round, participants proposed 18 variables potentially relevant for predicting PICM, spanning demographic, clinical, imaging, and device-related factors: Age at ablation, gender, history of heart failure, baseline LVEF, AF type, underlying cardiomyopathy, family history of cardiomyopathy, ischaemic heart disease, prior MI, prior CABG, LV dilation, intrinsic QRS duration, baseline LBBB, paced QRS duration, RV lead position (routinely assessed on fluoroscopy and postprocedural chest x-ray), non-CRT pacing system, RV pacing percentage before ablation, and at least moderate mitral regurgitation.

In the second round, structured voting categorized these into three priority groups. Priority 1 variables (≥75% agreement) included age, baseline LVEF, gender, baseline LBBB, intrinsic and paced QRS duration, RV lead position, history of heart failure, underlying cardiomyopathy, and non-CRT pacing system. Priority 2 variables (50–74% agreement) were ischaemic heart disease and type of AF. In comparison, Priority 3 (<50% agreement) included family history of cardiomyopathy, prior MI, prior CABG, LV dilatation, RV pacing percentage before AV node ablation, and mitral regurgitation.

In the third round, Priority 2 and 3 variables were re-evaluated. Ischaemic heart disease and permanent AF were confirmed for inclusion, while most Priority 3 variables were excluded, including family history of cardiomyopathy (80% exclusion), prior CABG, and mitral regurgitation. Prior MI received borderline support (60% inclusion) but was not prioritized.

Overall, consensus was reached on the most relevant predictors, including baseline LVEF, intrinsic and paced QRS duration, RV lead position, history of heart failure, and underlying cardiomyopathy, which were then validated in the retrospective cohort.

### Study population

Between January 2015 and December 2019, 658 patients underwent AV node ablation at Liverpool Heart and Chest Hospital (see Supplementary material online, *Figure S1*). Of these, 335 had CRT devices and were excluded, leaving 323 patients (49%) with non-CRT devices for analysis [median age, 75 years (IQR, 70–81); 57.0% female]. Baseline characteristics according to PICM status are summarized in *Table 1*. Patients with PICM were more likely to have broader intrinsic QRS [98 (86–107) ms vs. 90 (82–100) ms, *P* = 0.023], while other demographic and comorbidity profiles were comparable between groups.

**Table 1:** Baseline characteristics

Characteristic	PICM (*n* = 33)	No PICM (*n* = 290)	*P*-value
**Demographics**			
Age at procedure (years), median [IQR]	75 [72–79]	76 [70–80]	0.839
Female gender, *n* (%)	19 (57.6)	198 (68.3)	0.215
**Comorbidities**			
Dilated cardiomyopathy, *n* (%)	1 (3.2)	9 (3.5)	0.940
History of heart failure, *n* (%)	11 (33.3)	84 (29.0)	0.602
Ischaemic heart disease, *n* (%)	4 (12.1)	37 (12.8)	0.911
Prior myocardial infarction, *n* (%)	2 (6.1)	19 (6.6)	0.914
Prior revascularization, *n* (%)	2 (6.3)	24 (8.3)	0.682
Valvular heart disease, *n* (%)	5 (18.5)	39 (15.7)	0.700
**Electrocardiographic data**			
LBBB at baseline, *n* (%)	1 (3.0)	6 (2.1)	0.742
Intrinsic QRS duration, median [IQR]	98 [86–107]	90 [82–100]	0.023
Paced QRS duration, median [IQR]	162 [150–180]	156 [145–168]	0.059
**Cardiac imaging data**			
LVEF at baseline (%), median [IQR]	55 [55–60]	55 [50–60]	0.972
Moderate/severe mitral regurgitation, *n* (%)	4 (12.9)	36 (13.8)	0.885
LV dilation (0 vs. >0), *n* (%)	2 (6.5)	10 (3.9)	0.500
LVSD (0 vs. >0), *n* (%)	8 (24.2)	69 (25.7)	0.852
**Device-related data**			
RV lead position: Apical, *n* (%)	18 (56.3)	140 (55.1)	0.903
RV lead position: Septal, *n* (%)	11 (34.4)	106 (41.7)	0.425
RV lead position: Free wall, *n* (%)	3 (9.4)	8 (3.1)	0.084

Overall *n* varies by row due to missingness.

CABG, coronary artery bypass graft; LBBB, left bundle branch block; LV, left ventricle; LVSD, left ventricular systolic dysfunction; LVEF, left ventricular ejection fraction; PCI, percutaneous coronary intervention; PICM, pacing-induced cardiomyopathy; RV, right ventricle.

### Predictors of PICM

During a median follow-up of 192 days (IQR, 63–1003 days), 33 patients (9.8%) developed PICM, of whom 29 were classified as definite and 4 as likely PICM. The primary Cox model was based on this composite endpoint. Median time to PICM event was 387 days (IQR, 134–1191 days). On univariable analysis (*Table 2*), older age, longer intrinsic QRS, paced QRS duration, and free wall RV lead position were associated with PICM (*P* < 0.1). In the primary multivariable model (*Table 3*), free wall vs. septal RV lead position (HR 4.800, 95% CI 1.002–23.006, *P* = 0.0498) and intrinsic QRS duration (HR 1.350 per 10 ms, 95% CI 1.010–1.810, *P* = 0.042) remained independent predictors. Bootstrap internal validation of the multivariable Cox model showed modest performance. The apparent C-index was 0.646, and the optimism-corrected C-index was 0.613. The optimism-corrected calibration slope was 0.889, suggesting mild overfitting but overall reasonable internal stability (see Supplementary material online, *Tables S5A and B*). PICM occurred in three patients with free-wall RV lead position (10.7%) compared with eight patients without PICM (3.1%). Age and paced QRS duration did not retain their independent prognostic utility. Proportional hazards assumptions were satisfied for all covariates and globally (Schoenfeld test *P* = 0.52). Sensitivity analyses restricted to definite PICM cases showed consistent findings overall (*Tables 4–6*). In a parsimonious sensitivity model excluding paced QRS duration, both free wall vs. septal RV lead position and intrinsic QRS duration remained independently associated with definite PICM (*Table 7*). Notably, the association between RV free wall vs. septal lead position and PICM was highly fragile, as reclassification of only one PICM case from free wall to septal rendered the result non-significant in all tested scenarios.

**Table 2:** Univariate Cox regression analysis for predictors of pacing-induced cardiomyopathy

Variable	HR (95% CI)	*P*-value
**Demographics**		
*Age at procedure (years)*	*1.041* (*0.993–1.092)*	*0*.*098*
Female Gender	0.715 (0.358–1.427)	0.342
**Comorbidities**		
Heart failure history	1.349 (0.654–2.784)	0.418
Dilated cardiomyopathy (DCM)	0.613 (0.083–4.507)	0.631
Ischaemic heart disease	1.118 (0.393–3.185)	0.834
Prior myocardial infarction	1.711 (0.407–7.193)	0.463
Prior revascularization (PCI/CABG)	0.938 (0.224–3.93)	0.931
Prior CABG	0.846 (0.115–6.204)	0.869
Valvular heart disease	0.907 (0.342–2.405)	0.844
**Cardiac imaging data**		
LVEF (%) baseline	1.015 (0.966–1.066)	0.567
LVEDD	0.99 (0.951–1.03)	0.626
Degree of LV dilation	1.309 (0.764–2.242)	0.327
Mitral regurgitation	0.783 (0.273–2.246)	0.649
LV dilation	1.103 (0.262–4.644)	0.893
LVSD	1.236 (0.781–1.958)	0.366
LVSD	1.14 (0.514–2.529)	0.748
**Electrocardiographic data**		
*Intrinsic QRS duration (10 ms)*	*1.247* (*1.064–1.462)*	*0*.*006*
*Paced QRS duration (10 ms)*	*1.160* (*0.993–1.355)*	*0*.*061*
LBBB at baseline	1.497 (0.204–10.981)	0.692
**Device-related data**		
RV lead apical vs. septal	1.449 (0.684–3.068)	0.333
*RV lead free wall* vs. *septal*	*4.896* (*1.263–18.973)*	*0*.*022*

AF, atrial fibrillation; AVNA, atrioventricular node ablation; CABG, coronary artery bypass graft; CI, confidence interval; DCM, dilated cardiomyopathy; HR, hazard ratio; LBBB, left bundle branch block; LV, left ventricle; LVEDD, left ventricular end-diastolic diameter; LVEF, left ventricular ejection fraction; LVSD, left ventricular systolic dysfunction; PCI, percutaneous coronary intervention; PICM, pacing-induced cardiomyopathy; RV, right ventricle.

**Table 3:** Multivariate Cox regression analysis for predictors of pacing-induced cardiomyopathy

Variable	HR (95% CI)	*P*-value
Age at procedure (years)	1.093 (0.990–1.206)	0.077
RV lead position—free wall vs. septal	4.800 (1.002–23.006)	0.0498
Intrinsic QRS duration (10 ms)	1.350 (1.010–1.810)	0.042
Paced QRS duration (10 ms)	1.090 (0.750–1.590)	0.648

RV, right ventricle.

**Table 4:** Baseline characteristics in a sensitivity analysis, excluding patients without a complete set of initial investigations for LVEF decline and/or CRT upgrade follow-up, where PICM was still considered the most likely diagnosis

Characteristic	PICM (*n* = 29)	No PICM (*n* = 294)	*P*-value
**Demographics**			
Age at procedure (years)	75 [71–78]	76 [70–80]	0.602
Female gender, *n* (%)	17 (58.6)	200 (68.0)	0.303
**Comorbidities**			
Dilated cardiomyopathy, *n* (%)	0 (0.0)	10 (3.8)	0.302
History of heart failure, *n* (%)	9 (31.0)	86 (29.3)	0.841
Ischaemic heart disease, *n* (%)	3 (10.3)	38 (13.0)	0.686
Prior myocardial infarction, *n* (%)	2 (6.9)	19 (6.5)	0.928
Prior revascularization (PCI or CABG), *n* (%)	2 (6.9)	24 (8.2)	0.800
Prior PCI, *n* (%)	1 (3.4)	15 (5.2)	0.688
Prior CABG, *n* (%)	1 (3.4)	10 (3.4)	0.997
Valvular heart disease, *n* (%)	4 (17.4)	40 (15.8)	0.843
**Electrocardiographic data**			
LBBB at baseline, *n* (%)	1 (3.4)	6 (2.1)	0.641
Intrinsic QRS duration (ms)	98 [85–107]	90 [82–100]	0.065
Paced QRS duration (ms)	162 [146–184]	156 [144–168]	0.099
**Cardiac imaging data**			
LVEF at baseline (%)	55 [55–60]	55 [50–60]	0.862
LV dilation, *n* (%)	2 (7.4)	10 (3.8)	0.376
LVSD, *n* (%)	7 (24.1)	71 (26.1)	0.818
**Device-related data**			
RV lead position: Apical, *n* (%)	15 (53.6)	143 (55.4)	0.851
RV lead position: Septal, *n* (%)	10 (35.7)	107 (41.5)	0.556
RV lead position: Free wall, *n* (%)	3 (10.7)	8 (3.1)	0.047

Overall *n* varies by row due to missingness.

CABG, coronary artery bypass graft; LBBB, left bundle branch block; LV, left ventricle; LVSD, left ventricular systolic dysfunction; LVEF, left ventricular ejection fraction; PCI, percutaneous coronary intervention; PICM, pacing-induced cardiomyopathy; RV, right ventricle.

**Table 5:** Univariate Cox regression analysis for predictors of pacing-induced cardiomyopathy in a sensitivity analysis excluding patients without a complete set of initial investigations for LVEF decline and/or CRT upgrade follow-up, where PICM was still considered the most likely diagnosis

Variable	HR (95% CI)	*P*-value
**Demographics**		
Age at procedure (years)	1.033 (0.983–1.086)	0.199
Female Gender	0.736 (0.351–1.543)	0.418
**Comorbidities**		
Heart failure history	1.195 (0.544–2.626)	0.657
Dilated cardiomyopathy (DCM)	0.045 (0.000–75.343)	0.414
Ischaemic heart disease	0.954 (0.289–3.156)	0.939
Prior myocardial infarction	2.201 (0.519–9.336)	0.284
Prior revascularization (PCI/CABG)	1.116 (0.265–4.701)	0.881
Prior CABG	0.924 (0.126–6.799)	0.938
Valvular heart disease	0.839 (0.284–2.477)	0.750
Moderate/severe mitral regurgitation	0.657 (0.197–2.189)	0.494
**Cardiac imaging data**		
Baseline LVEF (%)	1.017 (0.964–1.072)	0.545
Baseline LVSD (combined)	1.276 (0.790–2.061)	0.319
LV dilation	1.298 (0.306–5.508)	0.724
LVSD	1.119 (0.478–2.621)	0.796
Baseline LVEDD (mm)	0.991 (0.951–1.032)	0.667
AF classification	1.028 (0.647–1.635)	0.907
**Electrocardiographic data**		
**Intrinsic QRS duration (ms)**	1.022 (1.004–1.039)	**0**.**013**
**Paced QRS duration (ms)**	1.015 (0.998–1.031)	**0**.**087**
**QRS duration difference (ms)**	0.986 (0.973–0.999)	**0**.**040**
LBBB at baseline	1.681 (0.228–12.388)	0.610
**Device-related data**		
RV lead position (apical)	1.092 (0.519–2.295)	0.817
RV lead position (septal)	0.665 (0.307–1.441)	0.301
**RV lead position (free wall)**	3.880 (1.142–13.185)	**0**.**030**
RV lead position (combined)	1.187 (0.619–2.279)	0.606
RV pacing % pre-AVNA	0.997 (0.983–1.011)	0.655

Significant predictors (*P* < 0.1) are bolded. Variables with *P* < 0.1 were included in the multivariate analyses.

AF, atrial fibrillation; AVNA, atrioventricular node ablation; CABG, coronary artery bypass graft; CI, confidence interval; DCM, dilated cardiomyopathy; HR, hazard ratio; LBBB, left bundle branch block; LV, left ventricle; LVEDD, left ventricular end-diastolic diameter; LVEF, left ventricular ejection fraction; LVSD, left ventricular systolic dysfunction; PCI, percutaneous coronary intervention; PICM, pacing-induced cardiomyopathy; RV, right ventricle.

**Table 6:** Multivariate Cox regression analysis for predictors of pacing-induced cardiomyopathy in a sensitivity analysis excluding patients without a complete set of initial investigations for LVEF decline and/or CRT upgrade follow-up, where PICM was still considered the most likely diagnosis

Variable	HR	95% CI	*P*-value
Age at procedure (years)	1.07	0.98–1.18	0.133
RV lead position—free wall vs. septal	4.92	1.02–23.60	0.047
Intrinsic QRS duration (10 ms)	1.35	1.00–1.81	0.051
Paced QRS duration (10 ms)	1.07	0.73–1.57	0.739

**Table 7:** Parsimonious multivariable Cox regression analysis of predictors of pacing-induced cardiomyopathy in a sensitivity analysis excluding patients with probable rather than definite PICM and excluding paced QRS duration

Variable	HR	95% CI	*P*-value
Age at procedure (years)	1.07	0.98–1.17	0.135
RV lead position—free wall vs. septal	5.58	1.38–22.52	0.016
Intrinsic QRS duration (10 ms)	1.38	1.06–1.80	0.018

### Exploratory intrinsic QRS cut-off analyses

**Figure 1:**
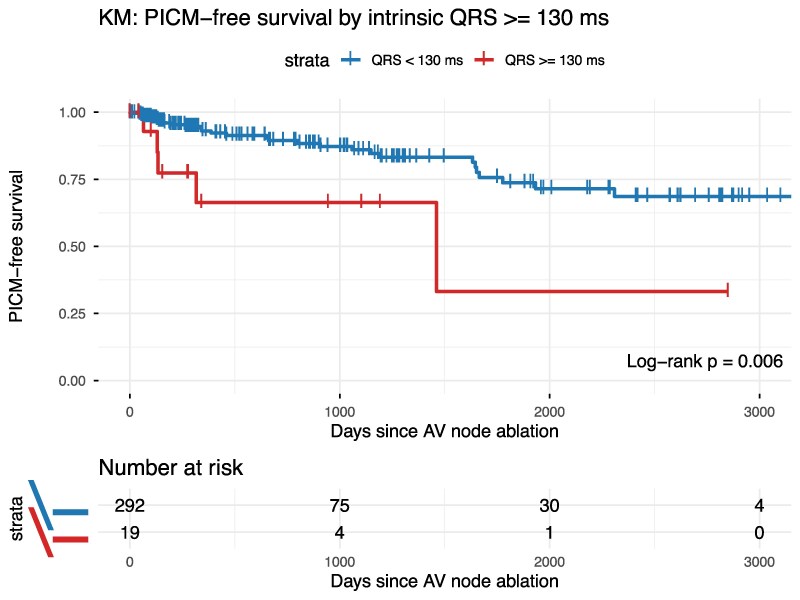
Pacing-induced cardiomyopathy (PICM)-free survival by intrinsic QRS ≥130 ms. Exploratory analysis suggests higher PICM risk for QRS ≥130 ms (HR 3.50, 95% CI 1.35–9.12, *P* = 0.01; log-rank *P* = 0.006). Findings should be interpreted with caution, given the small number of events.

Exploratory analyses of intrinsic QRS duration demonstrated that higher cut-offs were associated with progressively increased PICM risk (see Supplementary material online, *Table S1*). Prognostic accuracy metrics (sensitivity, specificity, PPV, NPV) were calculated for clinically relevant cut-offs ranging from to130 of 150 ms. A cut-off at 130 ms was strongly predictive (HR 3.504, 95% CI 1.350–9.120, *P* = 0.010; *Figure 1*). At this threshold, specificity was 95.0% (95% CI 91.8–97.0) and NPV 91.8% (95% CI 88.1–94.4), while sensitivity was 17.2% (95% CI 7.6–34.5) and PPV 26.3% (95% CI 11.8–48.8) (see Supplementary material online, *Table S2*).

At 135 ms, the HR increased further (HR 4.94, 95% CI 3.46–6.43, *P* = 0.004) with specificity 96.8% (95% CI 94.0–98.3) and PPV 30.8% (95% CI 12.7–57.6), though sensitivity declined to 13.8%. At 145 ms, risk was highest (HR 5.21, 95% CI 3.65–6.77, *P* = 0.008) and specificity reached 98.9% (95% CI 96.9–99.6), with a PPV of 50.0% (95% CI 18.8–81.2), but sensitivity was only 10.3%. Across thresholds, NPVs consistently exceeded 91%. When added to multivariable models (see Supplementary material online, *Table S3*), these cut-offs retained independent predictive value once age, paced QRS duration, and RV free wall vs. septal lead position were included.

### Pacing-induced cardiomyopathy, CRT upgrade, heart failure hospitalizations, and mortality

CRT upgrade was performed in 32 of 323 patients (9.9%) overall and in 25 of 33 patients (75.8%) with PICM. The composite endpoint of PICM and/or CRT upgrade occurred in 40 of 323 patients (12.4%). Heart failure hospitalization was more common among patients who developed PICM than among those who did not (21.2% vs. 1.4%, *P* < 0.001), whereas all-cause mortality was similar between groups (24.2% vs. 25.9%, *P* = 1.00). All-cause mortality was observed in 83 of 323 patients (25.7%). The composite of PICM, CRT upgrade, and/or death occurred in 113 of 323 patients (35.0%). In a multivariable Cox regression model including age, RV lead position (free wall), and intrinsic and paced QRS duration, only age remained independently associated with the composite endpoint of PICM, CRT upgrade, and/or death (see Supplementary material online, *Table S4*).

## Discussion

This study combined expert consensus and clinical validation to identify predictors of PICM in patients undergoing atrioventricular node ablation with non-CRT devices. Through three Delphi rounds, a high level of agreement was reached on the importance of variables such as baseline LVEF, intrinsic and paced QRS duration, RV lead position, history of heart failure, and underlying cardiomyopathy. Validation in a retrospective cohort confirmed that a free-wall RV lead position and longer intrinsic QRS duration were independent predictors of PICM, even in a model correcting for age and paced QRS. Findings were consistent in analyses restricted to definite PICM cases, supporting the robustness of the results. Exploratory analyses demonstrated that intrinsic QRS cut-offs, particularly those above 130 ms, identified subgroups at high risk of PICM, yielding high specificity, albeit sensitivity was low.

The integration of Delphi consensus with clinical validation provides insight into why some patients develop PICM after pace-and-ablate therapy. The expert panel prioritized variables already recognized as PICM predictors in the literature, such as baseline LVEF, intrinsic and paced QRS duration, and RV lead position^17,19,28–31^ and the Liverpool cohort confirmed their relevance. Longer paced QRS duration and free wall RV pacing likely reflect greater electrical dyssynchrony, quantifying the extent of pacing-induced conduction disturbance.^17,28–30,32^ In multivariable analyses, RV free wall position retained its prognostic utility, whereas paced QRS duration did not, possibly reflecting a shared mechanism of dyssynchrony for PICM. However, this association was highly fragile: in a sensitivity analysis repeating the Cox model after reclassifying free-wall PICM cases as septal, reclassification of a single case rendered the association non-significant. These observations align with the meta-analysis by Somma et al., which identified paced QRS duration and RV lead location as central correlates of PICM across RV-paced cohorts.^17^ The neutral results of prior RCTs comparing apical and septal pacing are reflected here.^17,33,34^ In our cohort, right ventricular apical pacing was not associated with a higher risk of PICM compared to septal pacing. The increased risk was confined to free-wall lead positions, indicating that this finding should not be extrapolated to apical pacing. While the higher risk with free-wall positions may indicate more pronounced pacing-induced dyssynchrony, it may also reflect the anatomical context in which these positions occur, including dilated ventricles with difficult septal implantation, which are themselves prone to mechanical delay.^17^

Intrinsic QRS duration exhibited a strong relationship with PICM risk. Longer intrinsic QRS duration was associated with increased risk in univariable and multivariable analyses. This pre-implant risk predictor is notable in the context of the neutral BIOPACE results, where routine BiVP offered no overall advantage over RVP, suggesting that CRT benefit may depend on identifying patients with markers of dyssynchrony rather than applying BiVP indiscriminately.^35^ Accordingly, the identification of intrinsic QRS cut-offs adds potential utility for patient selection for CRT. On univariable analyses, a threshold of 130 ms was associated with a 3.5-fold higher risk of PICM and maintained high specificity (95%). At 145 ms, specificity approached 99% and PPV reached 50%, albeit with sensitivity below 11%. These findings suggest that while such thresholds cannot be used to determine a low risk of PICM, they may identify subgroups in whom the risk is high and may support consideration of CRT in selected patients, pending prospective validation. These findings align with recent meta-analytic data demonstrating a mortality benefit of the pace-and-ablate strategy in heart failure, largely driven by CRT.^36^ In this context, our results extend current evidence by identifying patients at higher risk with conventional RV pacing, thereby supporting more refined selection and timely consideration of CRT. This aligns with current guidance, where the 2021 pacing guidelines provided only a class IIb, level C recommendation for BiVP in patients with preserved LVEF,^16^ and the 2025 EHRA CSP consensus did not endorse routine BiVP in this setting but stated that CSP may be appropriate, as it can reduce pacing-related dyssynchrony more directly.^37^ This is consistent with randomized data from CSPACE, where CSP showed more favourable remodelling and clinical outcomes than RV septal pacing in AV block patients.^38^ Similar results were demonstrated in a retrospective study of patients receiving LBBAP (including left ventricular septal pacing).^39^

From a practical perspective, this has implications in settings where CRT (including CSP and BivP) is not universally performed in pace-and-ablate treatment. In patients above these thresholds, referral to centres with CRT expertise should be considered and effort should be made to overcome barriers restricting access to CRT.^40^ Conversely, the low sensitivity of these intrinsic QRS cut-offs underscores that CRT should not be withheld in patients below the threshold if CRT implantation is an option and/or other risk factors or guideline indications are present. From a practical perspective, these findings may also inform leadless pacing selection strategies, supporting CRT or CSP in higher-risk patients and leadless pacing in lower-risk patients, pending prospective validation.

To our knowledge, this is the first study to employ a structured Delphi process for preselecting risk predictors for PICM in pace-and-ablate, ensuring that predictor testing is grounded in expert consensus across international centres. The large single-centre cohort, representing the highest reported number of pace-and-ablate patients without CRT to date, provided a uniform population with consistent implantation practices and routine follow-up. The systematic adjudication of outcomes, including the use of a gold standard diagnosis, further strengthened the final adjudication of the PICM endpoint.

## Limitations

This study has several limitations. Firstly, echocardiographic follow-up was performed as part of routine clinical care rather than at predefined intervals. This may introduce ascertainment bias, as symptomatic patients are more likely to undergo imaging, potentially inflating the observed event rate among those assessed. Conversely, late or asymptomatic cases of PICM may have been missed. In addition, the relatively short median follow-up of 192 days suggests that the true incidence of PICM is likely underestimated. The relatively short follow-up at the implanting centre partly reflects early discharge of a substantial proportion of patients to local care. However, clinically relevant cases, particularly those with suspected PICM or requiring CRT upgrade, would generally have been referred back to the implanting centre. Secondly, including likely PICM cases in the primary endpoint reflects real-world clinical practice but may introduce diagnostic uncertainty. However, sensitivity analyses restricted to definite PICM cases confirmed the robustness of the findings. Thirdly, the Delphi process was limited to participating European centres, potentially restricting the generalizability of expert consensus. The process was not fully anonymous, as structured feedback and exchange of ideas between rounds were permitted to refine variable selection. Fourthly, the RV lead position was determined using fluoroscopic and radiographic criteria rather than advanced imaging, which may have led to misclassification, particularly in distinguishing septal from free-wall positions using 2D imaging. No systematic use of CT or 3D imaging was performed. Fifthly, both the exploratory cut-off analyses and the multivariable model were based on a modest number of events. While bootstrap internal validation suggested reasonable internal stability, the optimism-corrected model performance was modest; accordingly, these findings should be regarded as exploratory and require external validation. Sixthly, post-ablation RV pacing burden was not systematically available in this retrospective dataset. Although near-complete ventricular pacing is generally expected after successful AV node ablation, this data was not collected. Seventhly, while PICM was chosen as the primary endpoint to ensure comparability with the existing literature, it serves as a surrogate for clinical outcomes. Hard endpoints, such as CRT upgrade, may be influenced by competing causes of LV dysfunction (e.g. intercurrent ischaemic events) and were therefore not used as the primary outcome, but are reported to provide additional clinical context. Eighthly, outcome adjudication was not blinded to exposure variables, which may introduce potential bias. Finally, the cohort included only patients undergoing AV node ablation with non-CRT devices. Findings may not apply to broader CIED populations, beyond pace-and-ablate patients.

### Conclusion

In patients undergoing AV node ablation with non-CRT devices, intrinsic QRS duration and RV free-wall lead position were independent predictors of pacing-induced cardiomyopathy. These findings, derived from a structured Delphi process and validated in a large cohort, support early risk stratification for upfront CRT implantation. Exploratory analyses of QRS thresholds should be considered hypothesis-generating and require external validation.

## Supplementary Material

oeag102_Supplementary_Data

## Data Availability

The data underlying this article will be shared on reasonable request to the corresponding author.
