# Supplementary material for: Predictors of pacing-induced cardiomyopathy in patients undergoing AV nodal ablation: insights from a Delphi process and retrospective cohort study
Source: Eur Heart J Open. 2026 Jun 29;6(3):oeag102. doi: 10.1093/ehjopen/oeag102 (PMC13312120; doi:10.1093/ehjopen/oeag102)
Supplement: oeag102_Supplementary_Data [file oeag102_supplementary_data.docx]

# **Supplemental Materials**

# **Figure S1.** Study flow diagram for cohort derivation and PICM ascertainment.

#
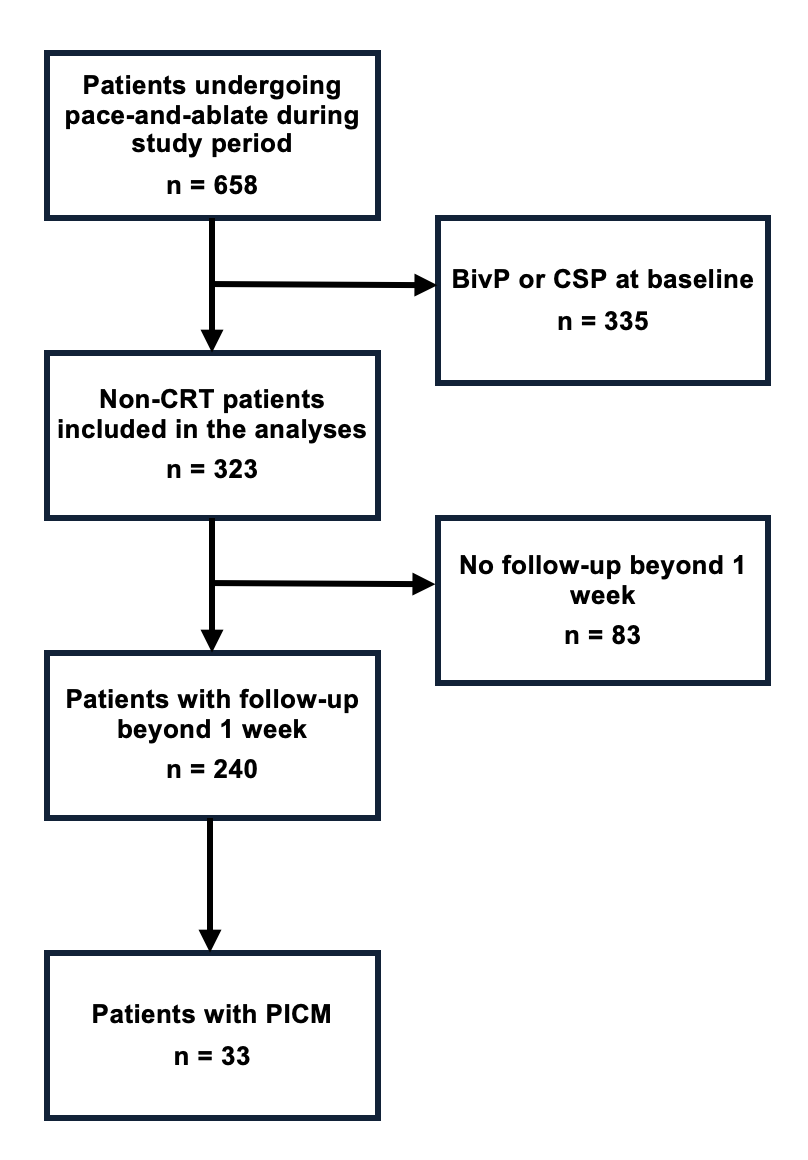


# Of 658 patients undergoing pace-and-ablate, 323 non-CRT patients formed the analytic cohort after exclusion of 335 with baseline CRT. After excluding 83 patients who died within 1 week or had no follow-up beyond 1 week, 240 remained for follow-up assessment, including 33 who developed PICM.

# **Table S1:** Cox regression for intrinsic QRS duration cut-offs and PICM risk.

| **QRS Cut-off (ms)** | **HR (95% CI)** | **p-value** |
| --- | --- | --- |
| 80 | 2.176 (0.660–7.140) | 0.199 |
| 85 | 1.732 (0.750–3.990) | 0.197 |
| 90 | 1.858 (0.880–3.910) | 0.102 |
| *95* | *2.158 (1.080–4.310)* | *0.029* |
| *100* | *2.736 (1.380–5.420)* | *0.004* |
| *105* | *2.456 (1.190–5.080)* | *0.015* |
| 110 | 1.846 (0.800–4.270) | 0.152 |
| 115 | 1.787 (0.730–4.350) | 0.201 |
| *120* | *2.452 (1.010–5.980)* | *0.049* |
| 125 | 2.552 (0.980–6.670) | 0.056 |
| ***130*** | ***3.504 (1.350–9.120)*** | ***0.010*** |
| ***135*** | ***4.320 (1.480–12.570)*** | ***0.007*** |
| ***140*** | ***4.334 (1.490–12.620)*** | ***0.007*** |
| ***145*** | ***4.650 (1.390–15.520)*** | ***0.012*** |
| ***150*** | ***4.516 (1.070–19.150)*** | ***0.041*** |
| 155 | 0.049 (—)* | 0.754 |
| 160 | 0.049 (—)* | 0.794 |

*Due to a low number of events in the group of QRS above the threshold, no 95% CI calculated.

# **Table S2.** Prognostic accuracy metrics by intrinsic QRS cut-off.

| **QRS Cut-off (ms)** | **Sensitivity, % (95% CI)** | **Specificity, % (95% CI)** | **PPV, %**  **(95% CI)** | **NPV, %**  **(95% CI)** |
| --- | --- | --- | --- | --- |
| 130 | 17.2% (7.6–34.5) | 95.0% (91.8–97.0) | 26.3% (11.8–48.8) | 91.8% (88.1–94.4) |
| 135 | 13.8% (5.5–30.6) | 96.8% (94.0–98.3) | 30.8% (12.7–57.6) | 91.6% (87.9–94.3) |
| 140 | 13.8% (5.5–30.6) | 97.2% (94.5–98.6) | 33.3% (13.8–60.9) | 91.6% (87.9–94.3) |
| 145 | 10.3% (3.6–26.4) | 98.9% (96.9–99.6) | 50.0% (18.8–81.2) | 91.5% (87.8–94.1) |
| 150 | 6.9% (1.9–22.0) | 99.3% (97.5–99.8) | 50.0% (15.0–85.0) | 91.2% (87.5–93.9) |

# **Table S3.** Multivariable Cox regression for PICM and QRS cut-offs:

**Intrinsic QRS ≥ 130 ms**

| **Variable** | **HR (95% CI)** | **p-value** |
| --- | --- | --- |
| Age at Procedure (per year) | 1.091 (0.982–1.211) | 0.105 |
| RV Lead Position – Free Wall vs Septal | 4.446 (0.959–20.615) | 0.057 |
| Paced QRS Duration (ms) | 1.016 (0.980–1.053) | 0.381 |
| Intrinsic QRS ≥ 130 ms | 5.211 (1.281–21.187) | 0.021 |

**Intrinsic QRS ≥ 135 ms**

| **Variable** | **HR (95% CI)** | **p-value** |
| --- | --- | --- |
| Age at Procedure (per year) | 1.093 (0.985–1.212) | 0.094 |
| RV Lead Position – Free Wall vs Septal | 4.873 (1.009–23.521) | 0.049 |
| Paced QRS Duration (ms) | 1.010 (0.975–1.046) | 0.574 |
| Intrinsic QRS ≥ 135 ms | 9.187 (1.339–63.013) | 0.024 |

**Intrinsic QRS ≥ 140 ms**

| **Variable** | **HR (95% CI)** | **p-value** |
| --- | --- | --- |
| Age at Procedure (per year) | 1.093 (0.985–1.212) | 0.094 |
| RV Lead Position – Free Wall vs Septal | 4.873 (1.009–23.521) | 0.049 |
| Paced QRS Duration (ms) | 1.010 (0.975–1.046) | 0.574 |
| Intrinsic QRS ≥ 140 ms | 9.187 (1.339–63.013) | 0.024 |

**Intrinsic QRS ≥ 145 ms**

| **Variable** | **HR (95% CI)** | **p-value** |
| --- | --- | --- |
| Age at Procedure (per year) | 1.093 (0.985–1.212) | 0.094 |
| RV Lead Position – Free Wall vs Septal | 4.873 (1.009–23.521) | 0.049 |
| Paced QRS Duration (ms) | 1.010 (0.975–1.046) | 0.574 |
| Intrinsic QRS ≥ 145 ms | 9.187 (1.339–63.013) | 0.024 |

**Intrinsic QRS ≥ 150 ms**

| **Variable** | **HR (95% CI)** | **p-value** |
| --- | --- | --- |
| Age at Procedure (per year) | 1.089 (0.983–1.206) | 0.102 |
| RV Lead Position – Free Wall vs Septal | 5.125 (1.070–24.542) | 0.041 |
| Paced QRS Duration (ms) | 1.008 (0.974–1.043) | 0.660 |
| Intrinsic QRS ≥ 150 ms | 13.253 (1.841–95.403) | 0.010 |

**Table S4.** Multivariable Cox regression analysis for the composite endpoint of PICM, CRT upgrade, and/or all-cause mortality

| **Variable** | **HR (95% CI)** | **p-value** |
| --- | --- | --- |
| Age at Procedure (years) | 1.055 (1.011–1.101) | 0.013 |
| RV Lead Position – Free Wall vs Septal | 1.515 (0.542–4.237) | 0.429 |
| Intrinsic QRS Duration (10 ms) | 1.145 (0.985–1.332) | 0.079 |
| Paced QRS Duration (10 ms) | 0.958 (0.835–1.099) | 0.544 |

**Table S5A.** Internal validation of the multivariable Cox regression model using bootstrap resampling

| **Metric** | **Value** |
| --- | --- |
| Apparent C-index | 0.646 |
| Mean optimism in C-index | 0.033 |
| **Optimism-corrected C-index** | **0.613** |
| Apparent calibration slope | 1.209 |
| Mean optimism in calibration slope | 0.320 |
| **Optimism-corrected calibration slope** | **0.889** |
| Bootstrap resamples | 300 |

Internal validation was performed using 300 bootstrap resamples. Model performance is summarised by the apparent and optimism-corrected C-index and calibration slope. The multivariable model included age at procedure, RV lead position, intrinsic QRS duration, and paced QRS duration.

**Table S5B.** Bootstrap-based coefficient stability of the multivariable Cox regression model

| **Variable** | **Apparent HR** | **Bootstrap mean HR** | **Bootstrap SD of coefficient** |
| --- | --- | --- | --- |
| Age at Procedure (years) | 1.055 | 1.056 | 0.025 |
| RV Lead Position – Free Wall vs Septal | 1.515 | 1.437 | 0.549 |
| Intrinsic QRS Duration (per 10 ms) | 1.145 | 1.149 | 0.081 |
| Paced QRS Duration (per 10 ms) | 0.958 | 0.943 | 0.082 |
